# Postoperative ERCP as proxy for clinically significant retained stones in a population-based cohort?^☆^

**DOI:** 10.1016/j.sopen.2026.01.007

**Published:** 2026-01-31

**Authors:** Eyvind Liljegren, Lina Hedestig, Emma Sverdén, Johanna Österberg, Lars Enochsson, Gabriel Sandblom

**Affiliations:** a Department of Clinical Science and Education Södersjukhuset, Karolinska Institutet, Sjukhusbacken 10, 118 83, Stockholm, Sweden; b Department of Urology, Karolinska University Hospital, Stockholm, Sweden; c Department of Surgery, Södersjukhuset, Stockholm, Sweden; d Department of Surgery, Mora hospital, Mora, Sweden; e Department of Diagnostics and Intervention, Surgery, Umeå University, Umeå, Sweden; f Department of Clinical Science, Interventions and Technology, Division of Orthopaedics and Biotechnology, Karolinska Institutet, Stockholm, Sweden

**Keywords:** Common bile duct stones, CBDS, Retained CBDS, Cholecystectomy

## Abstract

**Background:**

The rate of retained common bile duct stones (CBDS) following cholecystectomy can only be estimated if CBDS managed conservatively as well as CBDS treated with endoscopic retrograde cholangiopancreatography (ERCP) are identified. The aim was to explore the rate of retained CBDS and evaluate performance of ERCP as proxy for retained CBDS in a population-based setting.

**Methods:**

Data were collected from The Swedish Gallstone Surgery and Endoscopic Retrograde Cholangiopancreatography Register (GallRiks) on patients who underwent cholecystectomy 2015–2020 with suspected CBDS at South General Hospital, Stockholm, Sweden. Medical records were reviewed to identify rate of patients with events raising suspicion of passage of retained CBDS and compare this to the rate of ERCP for retained CBDS.

**Results:**

A total of 182 of 386 patients (47.2%) had CBDS on intraoperative cholangiography (IOC). During follow-up, 33 of the 182 presented with retained CBDS according to medical records. Of these, 24 had an ERCP registered in GallRiks with retained CBDS reported, whereas 9 had retained CBDS according to medical records only.

**Conclusion:**

Postoperative ERCP found valid as proxy for retained stones following surgery for CBDS and can be a quality measure for management of patients undergoing gallstone surgery with suspicion of CBDS.

## Graphical abstract

**Figure:**
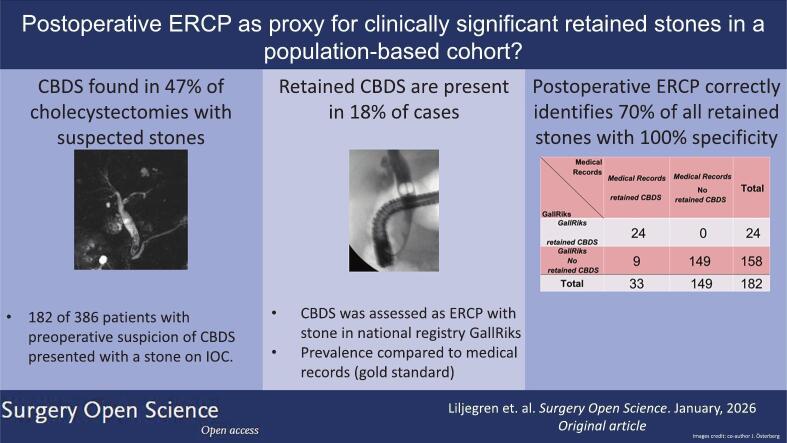

## Introduction

The management of patients with incidentally detected asymptomatic common bile duct stones (CBDS) remains a subject of ongoing international discussion. Central to this debate is the question of whether interventions such as endoscopic retrograde cholangio-pancreatography (ERCP) offer more benefit than the risk of overtreatment or iatrogenic complications, and whether it is prudent to treat asymptomatic CBDS conservatively. ERCP, while effective for stone removal [1], carries an inherent risk for post-ERCP pancreatitis (PEP), cholangitis, bowel perforation, bleeding, and other complications [2], [3]. Conversely, opting to leave an assumingly harmless gallstone *in situ* could lead to development of symptomatic CBDS, causing biliary pancreatitis, cholangitis, and potentially severe morbidity. Moreover, delayed detection of CBDS may necessitate emergency ERCP instead of planned pre- or intraoperative ERCP, which further complicates patient management [4]. In a Swedish healthcare setting, where routine intraoperative cholangiography (IOC) is recommended primarily for safety reasons [5], the rate of incidental CBDS is likely higher compared to settings where selective imaging only is performed.

The spontaneous clearance rate of CBDS has been shown to be relatively high, especially for small CBDS, raising the argument that asymptomatic CBDS should be left *in situ* [6], [7]. On the other hand, Möller et al. [8] found that the risk for an unfavourable outcome at 30 days follow-up, such as complications due to retained CBDS, was significantly higher in the group where CBDS were left *in situ* compared to the group where measures to remove the CBDS were taken (25% *vs* 13% resp.).

Furthermore, in another Swedish population-based study on patients undergoing cholecystectomy, the risk for having to undergo an unplanned postoperative ERCP, assumed to be due to a retained CBDS, was significantly higher for patients managed conservatively compared to those undergoing intraoperative stone removal [9]. The latter was true even for the smallest asymptomatic CBDSs identified incidentally by IOC. This study had a longer follow-up but used a proxy measure to estimate the rate of retained CBDS that has yet to be validated. The rate of postoperative ERCPs registered in the population at large has not previously been correlated to actual retained CBDS prompting the intervention.

A small trial found rates of retained CBDS to be 15–42% [1] depending on removal method, but there is a scarcity of large follow-up studies assessing the rate of retained CBDS, partially due to the lack of comprehensive quality registers with complete long-term follow-up. However, data on whether an ERCP was performed or not may be more readily available for analysis. The extent to which an ERCP carried out postoperatively reflects the true rate of retained common bile duct stone in retrospective studies is not known.

The safest and most effective way of managing patients with asymptomatic CBDS has not been established, and the rate of retained CBDS after cholecystectomy is an important outcome measure that enables comparison of management strategies and their long-term efficacy. Whether postoperative ERCP is a valid proxy for clinically relevant retained CBDS is not known.

### Aims

The aim of this study based on a population-based register, was to investigate if postoperative ERCP showing CBDS may be used as proxy measure for clinically relevant retained CBDS. The ERCP rate from the GallRiks register was compared with data from the medical records which in this setting was used as a gold standard indicating retained CBDS. Furthermore, the aim was to explore the rate of clinically relevant retained CBDS in a surgical population at South General Hospital in Stockholm, Sweden, selected for high risk of CBDS at time of cholecystectomy.

## Materials and methods

### Study design

This study was conducted as a register-based cohort study comparing data in GallRiks with medical records to determine the rate of retained stones and how this relates to the frequency of events requiring ERCP.

### Selection of patients and collection of data

The Swedish Gallstone Surgery and Endoscopic Retrograde Cholangiopancreatography Register (GallRiks), covers more than 90% of all cholecystectomies and ERCPs performed in Sweden since 2009 [10], [11], [12].

A request to access and review medical records was submitted after approval by the Swedish Ethics Review Authority, (Dnr 2022-03269-01). Gallriks utilises an opt out alternative for any patient who does not accept the entry of data into to registry and information on how to opt out is provided to the patient by the caregiver.

Data were collected by searching GallRiks for patients who had undergone cholecystectomy at South General Hospital, Stockholm, between 2015 and 2020 and who were registered as suspicion of CBDS being the indication for surgery. Complete medical records for the patients in the cohort were retrieved, pseudonymised, and saved in a separate database. Data were collected September 2022 and analysed and coded between September and November 2022. Data on all ERCPs performed on the patients in this cohort were assembled from GallRiks until September 2022. Follow-up time from cholecystectomy was 20 to 80 months. A review of the patient's medical records was carried out by two assessors (LH and EL) blinded to late events registered in GallRiks.

### Setting up the database from medical records

**Fig. 1:**
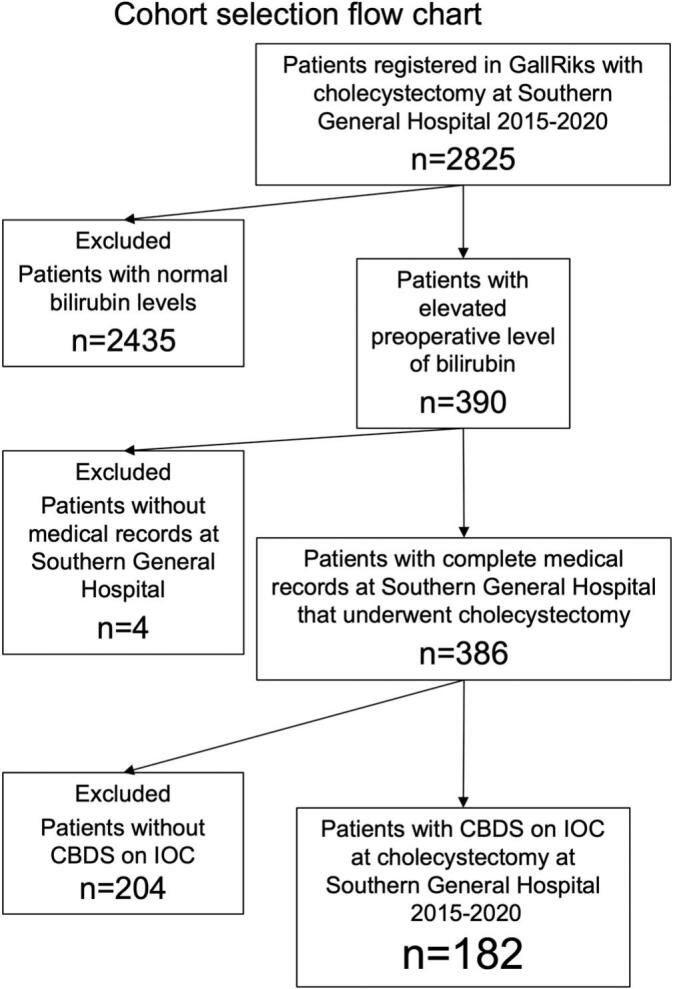
Cohort selection flow chart. CBDS = Common bile duct stones, IOC = intraoperative cholangiography.

A cohort was selected by searching the GallRiks database for patients that underwent cholecystectomy at South General Hospital, Stockholm, Sweden between January 1st 2015 and December 31st 2020. Exclusions were made according to the flow diagram in Fig. 1.

### Criteria for coding for retained CBDS in the medical records review

**Table:** 

Variable	Description
Elevated bilirubin levels (yes/no)	If medical records stated elevated bilirubin levels (above 30 μmol/L) or clinical signs of jaundice.
Abdominal Pain (yes/no)	Yes, if patients had any kind of abdominal pain.
Verified CBDS on imaging (yes/no)	Yes if, verified CBDS on Computed Tomography (CT), abdominal Ultrasound (US) or Magnetic Resonance Cholangio Pancreatography (MRCP).
Date of retained CBDS (clinical)	Date of first contact with healthcare due to clinical signs of CBDS (elevated bilirubin levels in combination with abdominal pain or verified CBDS on imaging)
Diagnosis CBDS (yes/no)	Yes if, CBDS was diagnosed in medical records.
Postoperative ERCP performed? (yes/no)	Yes if, ERCP was performed more than 7 days after the cholecystectomy.
Finding of retained CBDS on ERCP? (yes/no)	Yes, if findings of a retained CBDS at ERCP were documented in medical records.
Date of retained CBDS (ERCP)	Date of postoperative ERCP performed with retained CBDS.

Patients with either clinical, (elevated bilirubin levels in combination with abdominal pain), or radiological (a verified CBDS on imaging or ERCP) signs of a retained CBDS, were categorised as having a retained CBDS. Furthermore, any ERCPs performed within 7 days after the cholecystectomy were assumed to be part of a primary two-stage management and not considered to be due to suspected retained CBDS.

#### Postoperative ERCP as proxy for long-term retained CBDS based on GallRiks data

Postoperative ERCPs were identified in GallRiks during the entire follow-up period of 20 to 80 months after cholecystectomy. If an ERCP was carried out that reported CBDS during follow-up, the patient was categorised as having a retained CBDS. Data were compiled and structured using R using R studios (R Core Team (2023). _R: A Language and Environment for Statistical Computing_. R Foundation for Statistical Computing, Vienna, Austria. <https://www.R-project.org/>. in R studios, Version 2023.12.0+369 (2023.12.0+369)).

Sensitivity as well as specificity of the proxy when compared to CBDS noted in the medical records were calculated by cross-tabulation.

### Ethics approval

This study collected sensitive personal data and was subject to the Swedish ethics review act, 3 § 1. An ethics application was submitted and approved by Swedish Ethics Review Authority in June 2022 (Dnr. 2022-03269-01).

## Results

After exclusion of four patients due to missing medical records, a total of 386 patients were included for analysis of data from the medical records. No patient with ASA-classification ≥ IV was identified. Altogether 179 of the patients were males and 207 females. Median age was 52 years (range 15–89 years). *Circa* 50% of the patients included had ASA-classification II (mild systemic disease) (Table 1).

**Table 1:** Patient characteristics and proportions of retained common bile duct stones according to medical records and postoperative ERCP.

	Patient characteristics		
	Retained CBDS according to medical records		
	Total (*n* = 182)	No (*n* = 149)	Yes (*n* = 33)
Sex			
Female	114 (62.6%)	95 (63.8%)	19
Male	68 (37.4%)	54 (36.2%)	14

Age (years)			
Median (IQR)	50.5 (29.0)	49.0 (30.0)	54.0

ASA classification			
ASA I	58 (31.9%)	48	10
ASA II	92 (50.5%)	77 (51.7%)	15
ASA III	32	24	8

CBDS management at cholecystectomy^a^			
No measure to clear	1	0	1
Peroperative ERCP	166 (91.2%)	136 (91.3%)	30
Flushed out during IOC	9	8	1
Transcystic extraction	6	5	1

Retained CBDS at postop ERCP			
No	158 (86.8%)	149 (100.0%)	9
Yes	24	0	24

ASA = American Society of Anesthesiologists, CBDS = Common bile duct stone, ERCP = Endoscopic retrograde cholangiopancreatography, IOC = Intraoperative cholangiography.

a 43 cases with missing data in GallRiks were completed with data from medical records (Flush/manipulate had one case, Transcystic had two, the other 40 cases were peroperative ERCP).

Of the cohort of 386 patients, 182 (47.2%) presented with a CBDS at IOC during cholecystectomy and there was a slight overrepresentation of females who had a CBDS (114 *versus* 68).

### Retained CBDS according to the medical records

A total of 182 of the 386 patients (47.2%) included had CBDS on IOC. These patients were followed 20 to 80 months. Of those with CBDS at IOC, 33 presented with retained CBDS in the follow-up period according to the medical records. None was diagnosed within seven days of cholecystectomy. Of these 33 patients, 24 had an ERCP registered in GallRiks with retained CBDS, whereas a further 9 had retained CBDS that was only reported in the medical records. Of the nine patients with retained CBDS identified in the medical records only, four had spontaneous passage after diagnosis and five had an ERCP that was not correctly registered in GallRiks as identifying retained CBDS, even when it was reported in the medical records.

Comparison was made between ERCPs registered as showing CBDS after cholecystectomy (as proxy) and retained CBDS reported in the medical records (as gold standard), and sensitivity and specificity were calculated as shown in Table 2.

**Table 2:** Sensitivity and specificity of retained CBDS at ERCP compared to retained CBDS according to medical records.

	Medical records Retained CBDS	Medical records No retained CBDS	Total
GallRiks			
CBDS at ERCP	24	0	24
GallRiks No CBDS at ERCP	9	149	158
Total	33	149	182
	Sensitivity: 72.7%	Specificity: 100%	

CBDS: Common bile duct stone.

ERCP: Endoscopic Retrograde Cholangiopancreatography.

## Discussion

The present study demonstrated an 18.1% cumulative incidence of retained CBDS after cholecystectomy in a cohort with high predicted risk for CBDS preoperatively and who were found to have CBDS at IOC. Postoperative ERCP for stone had a 72.7% sensitivity and 100% specificity for retained CBDS compared to data from the medical records. The discrepancy between the incidence of postoperative retained CBDS presenting with clinical symptoms and the incidence of ERCP showing retained stones, was moderate. Postoperative ERCP is thus a relevant and valid proxy measure for clinically relevant retained stones when assessing the risk for retained stones at the population level.

In the cholecystectomy community, there is an ongoing international discourse on how to manage patients with CBDS. While active intervention to extract stones intraoperatively at cholecystectomy reduce the rate of retained CBDS [8], [9], the rate of retained CBDS varies in different estimates and understanding their natural course is crucial when evaluating management strategies. Contrary to many other countries, the routine in Sweden is to manage patients with CBDS by cholecystectomy with intraoperative ERCP rather than preoperative ERCP followed by cholecystectomy.

The present cohort was selected based on the preoperative high likelihood of CBDS and had a high rate of CBDS at IOC (47.2%) compared to previous studies with reported prevalence of about 10–20% [13]. The rate of retained CBDS among those with a positive IOC was higher (18.1%) than previous studies with rates of retained CBDS between 2.3% [14] and 9.2% [9], [15], [16]. However, in another randomised trial on cases with confirmed CBDS at cholecystectomy, the rate of retained CBDS was 15–42% after attempted removal with ERCP or laparoscopic bile duct exploration, which is more in line with the present study [1].

This study demonstrated the sensitivity and specificity of postoperatively performed ERCP as proxy for retained CBDS to be 72.7% and 100% respectively, compared to CBDS registered in the medical records as gold standard. This proxy measure for retained CBDS is therefore on par with the sensitivity of conventional computed tomography (CT) at 69–87% and the specificity of magnetic resonance cholangio pancreatography (MRCP) at 96% [13].

ERCPs not correctly registered in GallRiks contributed to five cases of retained CBDS not included as proxy. This is consistent with previous validation studies of GallRiks with 10% of all gallstone interventions in Sweden that are not registered in GallRiks [11]. In addition, the study was limited to patients managed at South General Hospital. Thus, any patient that had an ERCP anywhere else despite having had their cholecystectomy and following medical records at South General Hospital, would not be identified using the GallRiks criteria in this study and thus reduce the estimated sensitivity. It is unlikely that this was the cause of loss to follow-up because it is customary to refer patients, when feasible, to the centre responsible for initial treatment. In one case, a patient that was referred to us from Örebro, a town 200 km from Stockholm, with retained CBDS following surgery at our centre.

Four patients with clinical signs of retained CBDS did not undergo postoperative ERCP, due either to spontaneous passage or frailty or high comorbidity. ERCP is an invasive treatment and patients that are unsuitable for invasive treatment may receive symptomatic treatment only, such as biliary drainage and stenting [17]. However, according to medical records no patient in this cohort was explicitly excluded from ERCP because of frailty or expected not to survive the procedure.

### Strengths and limitations

One of the strengths of the study is the use of GallRiks as a data source. GallRiks is a large and validated register that covers about 90% of all cholecystectomies and ERCPs performed in Sweden [12]. However, patients with retained CBDS at hospitals other than South General Hospital were not included which may lead to both under- and overestimation of the sensitivity and specificity.

Another possible limitation is the risk for incorrect inclusion of patients with non-specific abdominal pain and elevated bilirubin levels as having retained CBDS. This may lead to inaccurate inclusion of patients with pancreatitis or other gastrointestinal conditions who present with similar symptoms that may not be related to CBDS. To mitigate misclassification, questionable cases were reviewed by more experienced surgeons in the research group to judge the most likely underlying diagnosis.

In this study, the cohort was selected from patients who were suspected of having CBDS before undergoing surgery, primarily for feasibility reasons to reduce the number of medical records to be reviewed. It is important to note that routine intraoperative cholangiography (IOC) is performed in approximately 90% of cholecystectomy cases in Sweden, regardless of whether patients have symptoms of CBDS or not. Therefore, the rate of IOC utilisation should not be influenced by the symptoms of patients undergoing cholecystectomy.

Although we can reasonably assume that the rate of undetected retained stones in the GallRiks register is similar between incidentally detected and preoperatively suspected CBDS, suggesting robust sensitivity of the proxy, caution is still warranted when interpreting the results of this study, especially concerning the management of incidental common bile duct stones.

Furthermore, these results are assembled in Sweden, where the healthcare system is relatively homogeneous, there are few private units, and the routines for handling patients with common bile duct stones are standardised. The results should be interpreted with care when extrapolating them to healthcare systems with more heterogeneous populations.

## Conclusions

In conclusion, this cohort study demonstrated a rate of 47.2% common bile duct stones at cholecystectomy among patients with preoperative suspicion of CBDS, with an 18.1% rate of retained stone after surgery or any stone clearing measures deemed appropriate. Postoperative ERCP with finding of CBDS as a proxy for retained stone identified 72.7% of all patients with retained CBDS, while excluding 100% of all patients without one, compared to data from the medical records. This study suggests that postoperative (not within 7 days) ERCP is a suitable proxy to estimate retained CBDS and that few patients with retained CBDS are left without treatment. This may, however, somewhat underestimate the true rate compared to retained CBDS noted in the records.

## CRediT authorship contribution statement

**Eyvind Liljegren:** Writing – review & editing, Writing – original draft, Software, Project administration, Methodology, Formal analysis, Data curation, Conceptualization. **Lina Hedestig:** Investigation, Data curation. **Emma Sverdén:** Writing – review & editing, Supervision, Methodology, Conceptualization. **Johanna Österberg:** Writing – review & editing, Supervision, Methodology, Conceptualization. **Lars Enochsson:** Writing – review & editing, Supervision, Methodology. **Gabriel Sandblom:** Writing – review & editing, Supervision, Resources, Project administration, Methodology, Funding acquisition, Formal analysis, Conceptualization.

## Ethics approval

Ethics approval was granted by Swedish Ethics Review Authority, (Dnr 2022-03269-01).

## Declaration of competing interest

Eyvind Liljegren: No conflicts of interest to declare

Lina Hedestig: No conflicts of interest to declare

Emma Sverdén: No conflicts of interest to declare

Lars Enochsson: No conflicts of interest to declare

Johanna Österberg: Lectures and workshops: For Ethicon, Baxter and Mölnlycke. Has no relation to this article. Payments for 3 single lectures/workshops past 36 months.

Board member in Swedish Register for Gallstone Surgery and ERCP (GallRiks)Unpaid

Board member in Swedish Register for (Inguinal) Hernia Surgery (Sv Bråckregistret), Unpaid

Gabriel Sandblom: Board member in Swedish Register for Gallstone Surgery and ERCP (GallRiks)Unpaid
